# Backward Flux Re-Deposition Patterns during Multi-Spot Laser Ablation of Stainless Steel with Picosecond and Femtosecond Pulses in Air

**DOI:** 10.3390/ma14092243

**Published:** 2021-04-27

**Authors:** Tong Zhou, Sebastian Kraft, Walter Perrie, Jörg Schille, Udo Löschner, Stuart Edwardson, Geoff Dearden

**Affiliations:** 1 Laser Group, School of Engineering, University of Liverpool, Brownlow Street, Liverpool L69 3GH, UK; pstzhou4@liverpool.ac.uk (T.Z.); me0u5040@liverpool.ac.uk (S.E.); gdearden@liverpool.ac.uk (G.D.); 2 Laserinstitut Hochschule Mittweida, Hochschule Mittweida University of Applied Sciences, Technikumplatz 17, 09648 Mittweida, Germany; kraft@hs-mittweida.de (S.K.); schille@hs-mittweida.de (J.S.); loeschne@hs-mittweida.de (U.L.)

**Keywords:** laser ablation, ultrafast, re-deposition

## Abstract

We report on novel observations of directed re-deposition of ablation debris during the ultrafast laser micro-structuring of stainless steel in the air with multi-beams in close proximity on the surface. This interesting phenomenon is observed with both 10 ps and 600 fs NIR laser pulses at 5 kHz repetition rate. Ablation spot geometries could be altered with the use of beam splitting optics or a phase-only Spatial Light modulator. At low fluence (F ~ 1.0 J cm^−2^) and pulse exposure of a few hundred pulses, the debris appears as concentrated narrow “filaments” connecting the ablation spots, while at higher fluence, (F ~ 5.0 J cm^−2^) energetic jets of material emanated symmetrically along the axes of symmetry, depositing debris well beyond the typical re-deposition radius with a single spot. Patterns of backward re-deposition of debris to the surface are likely connected with the colliding shock waves and plasma plumes with the ambient air causing stagnation when the spots are in close proximity. The 2D surface debris patterns are indicative of the complex 3D interactions involved over wide timescales during ablation from picoseconds to microseconds.

## 1. Introduction

The interaction of intense ultrafast laser radiation with atoms, gases and solid materials has led to remarkable scientific advances such as High Harmonic Generation for attosecond spectroscopy [1,2], the use of filamentation for atmospheric probing [3,4], two-photon microscopy [5,6] and intraocular fs-LASIK in eye surgery [7]. In particular, laser ablation (LA) of materials with ps and sub-ps temporal pulse length has advanced materials analysis in areas such as Laser Ablation Inductively Coupled Mass Spectrometry (LA-ICP-MS) [8,9,10] and Laser-Induced Breakdown Spectroscopy (LIBS) [11,12]. With temporal pulse lengths <10 ps in ultrafast laser ablation, the instantaneous energy deposition reduces plasma absorption while minimizing melt and thermal diffusion during the pulse and yield high precision material removal [13,14]. This allows for easy generation of plasmonic, interferometric and hierarchical microstructures on, for example, stainless steel [15,16,17]. In metals, which have absorption coefficients typically α ~10^6^ cm^−1^, a thin layer with thickness d ~10–30 nm is converted to a plasma at a solid density which expands well after the pulse is absorbed [18,19].

In vacuo, laser ablation can be described as a free expansion [20,21,22,23] while the laser plume which develops in the presence of an environmental gas is physically much more complex involving timescales from picosecond to nanosecond and microsecond. After electron heating during the pulse, the lattice heats typically over a few picoseconds due to e-phonon coupling, raising the surface temperature well above the evaporation point. This results in fast surface electron emission due to thermionic [24] or multi-photon absorption [25,26] and a remarkable early stage “air plasma” can develop during the first 150 ps [27,28], with electron density n_e_ ~ 10^20^ cm^−3^ and a transient electric field developing above the surface [29].

On the nanosecond timescale in air, the plasma plume expands rapidly and initially longitudinally (at speed v ~ 10^6^ cm s^−1^), colliding with the high density of surrounding gas molecules, (N_1atm_ ~ 2.6 × 10^19^ cm^−3^), confining the plasma to the surface region while creating a blast (shock) wave which moves ahead of the plume [30,31,32,33,34]. As the longitudinal expansion decelerates rapidly, the plasma expands laterally after a few ×100 ns (v_lat_ ~ 10^5^ cm s^−1^) and can form a vortex structure with plasma plume concentrated at the ablation spot edge [35]. Transient electron temperatures in the plasma can reach T_e_ >> 10^4^ K [24], then plasma cooling (over a few hundreds of ns to µs) occurs due to plasma expansion, electron-ion recombination and collisions with the background gas molecules. When thermal equilibrium is achieved in the plasma (~100 ns), this is the regime for LIBS and material analysis with fluorescence from highly excited atoms/ions [33,34]. As the plasma cools further, molecular species are formed at later times (~2–50 µs) due to atomic collisions, recombination and oxidation occur after the shock wave collapses [36]. Aerosols and nanoclusters are generated during a nucleation-condensation process with nanoparticle agglomerates re-deposited symmetrically by backward flux around a single spot [35,37]. While time-resolved spectral information using gated ICCDs can help elucidate the plasma dynamics, time-resolved scattering can detect nanoparticle formation and re-deposition on the microsecond timescale [35].

Debris re-deposition was first investigated during UV, excimer micro-structuring of polymers (e.g., Polyimide, PI), responsible for the developing cone structure with debris field dependent on spot shape, inferring strong horizontal forces close to the substrate surface [38]. Apparent field rotations with non-spherical spots were numerically modelled as a purely gas dynamic effect [39]. The debris radius was also shown to follow blast wave theory during excimer ablation of polyimide in air and inert gases [40]. Time-delayed (20–80 µs delay) weak emission detected at the plume periphery consisted mainly of continuum radiation consistent with thermal radiation from solid particles [20]. The difference in ablation geometries observed with a linear array of closely spaced ablation spots inferred stronger plume interactions in the middle [41]. Monte Carlo simulation of the expansion of a copper plasma in the presence of a background gas (Ar) shows compression of the ambient gas atoms by the plume (snowplough effect) for pressures >50 Pa and narrowing of the plume with increasing pressure while predicting backward plume motion and vortical flow at the plume periphery [42]. In the case of excimer ablation of stainless steel in air, (F = 10 J cm^−2^/308 nm/25 ns) time-resolved ICCD images of the ablation plume up to 10 µs delay show this developing vortical structure after 1 µs and fully developed at 10 µs delay with a high density of nanoparticles deposited at the periphery [35]. Time-resolved light scattering during ablation also demonstrated that debris re-deposition occurred over a time delay of 1–60 µs after the pulse.

Polymers (such as PI, a photoresist) have strong absorption coefficients α > 10^4^ cm^−1^ in the UV leading to electronic excitation and “cold” ablation via direct bond breaking [43]. In the air, a plasma plume, carbonization (thermal component) and re-deposition around the ablation spot occurs but can be minimized using, for example, H_2_ as a reactive, ambient gas [44]. Low ablation thresholds F_th_ < 50 m J cm^−2^ and precise etch rates from 25 nm/pulse are observed [45]. On steel, UV (248 nm/25 ns) ablation re-deposition generates iron oxide nanoparticles with a wide distribution φ = 2–20 nm while at 500 fs/248 nm, this narrows to φ = 3 ± 2 nm [46]. The use of NIR, ns pulses result in the incorporation of nitrogen, as well into the re-deposited layer [47].

More recently, there has been an emphasis on temporal plume expansion with bursts of ultrashort pulses [48], and with a very high repetition rate USP Lasers [49,50] driven by the requirement to increase material ablation rates through a better understanding of the plume expansion and plasma absorption by the following pulses.

The interaction dynamics of shock waves during fs laser multi-spot ablation at a water/air interface were recently observed using transient reflection creating colliding supersonic airflows [51,52]. The authors point to the potential for producing focussed ablation plumes relevant to pulsed laser thin film deposition [53,54]. This idea has been investigated here using the observed backward re-deposition patterns from multi-spot ultrafast laser ablation on stainless steel as a novel approach highlighting the resulting plasma plume and shock wave interactions. Symmetric jets, with debris ejected at right angles to the spot axis are reported for the first time, and we present a tentative explanation of the physics behind these observations. The results may well impact the crucial area of Laser-Induced Forward Transfer of nanoparticle thin films at ambient pressure [55,56].

## 2. Materials and Methods

**Figure 1:**
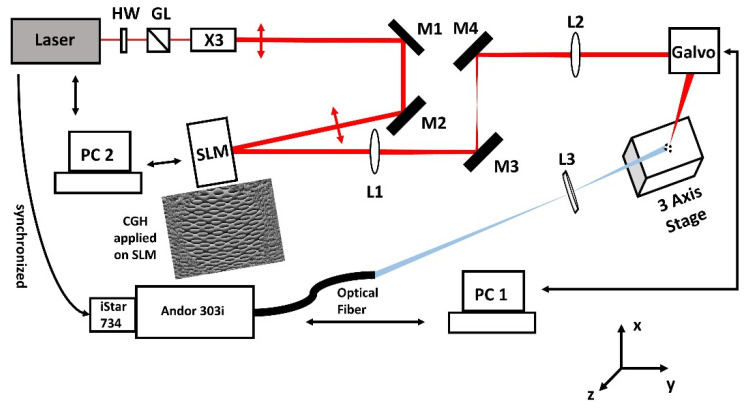
Optical set-up for 10 ps/5 kHz multi-spot ablation of stainless steel in the air. An attenuated and expanded beam from a Nd:Van Regen amplifier is directed to the phase-only SLM at low AOI then imaged via a 4f optical system (f = 400 mm) to the galvo input aperture. Appropriate phase-only CGH’s could create arbitrary spot geometries and separations at the steel surface. A time-resolved spectrometer with triggered ICCD (synchronised to Regen amplifier) allowed measurement of the spectral plasma dynamics by focusing the plasma emission on a fibre coupler.

Experiments were carried out with two separate laser and optical systems based at the Universities of Liverpool and Mittweida where the temporal pulse lengths available were 10 ps and 600 fs, respectively. Figure 1 shows a schematic of the optical system in Liverpool. The output from a Nd:VAN seeded Regenerative amplifier (High-Q IC-355-800 ps, 10 ps/1064 nm Photonic Solutions Ltd., Edinburgh, UK) was attenuated, expanded (×3), then directed at low AOI to a phase-only Spatial Light Modulator (SLM, Hamamatsu-10468-03, Hamamatsu Photonics, Hamamatsu, Japan) for generating multi-spot patterns and focussed to a sample mounted on a three-axis stage able to bring the substrate surface to the focal plane. Computer Generated Holograms (CGHs) controlling spot numbers and separation on the substrate were generated using Inverse Fourier Transforms in the Hamamatsu software, while a pick-off optic (not shown) could direct spot patterns to a CCD camera prior to multi-spot ablation. This allows precise electronic control of spot separation with no mechanical movements. A 4f optical system (f_1_ = f_2_ = 400 mm) relayed the modulated beam from the SLM (addressed with CGHs) to the input aperture of a galvo system which directed the beams to the target surface, focussed by an f-theta lens (f = 100 mm). A fast-mechanical shutter (Thorlabs SH05, Thorlabs Ltd., Ely, UK) allowed the pulse number on target to be varied, synchronised to the scanning software (SCAPS GmbH). The expanding plumes were imaged to a time-resolved spectrometer (Andor Shamrock, model SR303i with intensified CCD, iStar 734, 2 ns gate width, Andor Technology Ltd., Belfast, UK) and synchronised from the 5 kHz Regenerative amplifier.

**Figure 2:**
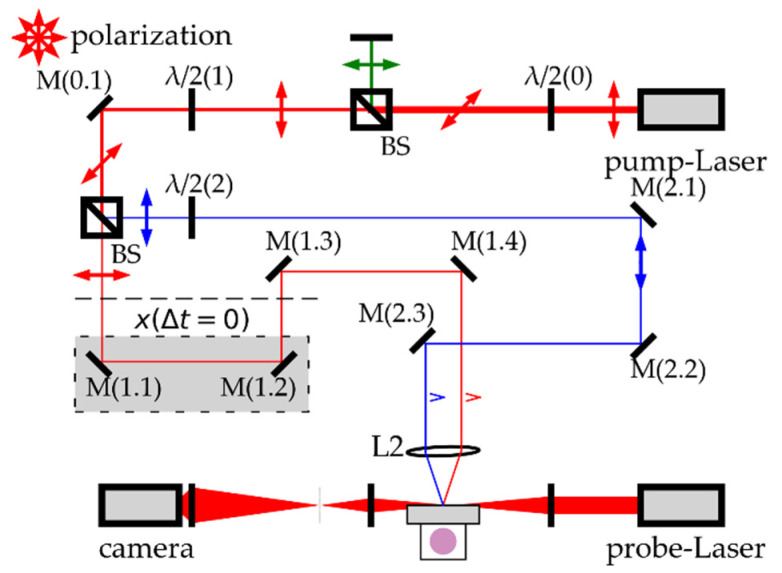
Scheme of the optical set-up for double spot ablation with simultaneous shadowgraphy of stainless steel in the air. The pump laser, probe laser and axis and shadowgraph capture were electrically synchronised. The attenuated and split pump beams are focussed and temporally overlapped at the sample surface by one optic lens L2. The temporal (by moving M (1.1) and M (1.2)) and geometrical adjustment (by tilting M (1.4)) can be monitored with a spot monitor in the focal plane.

Figure 2 shows a schematic of the optical system for 600 fs/5 kHz double spot ablation (Mittweida). The pump laser (FX-Series, edgewave GmbH, 1030 nm/600 fs, Würselen, Germany) output was attenuated by HWP/polarising beam splitter BS. Afterwards, a second HWP/PBS combination produce two optical lines (1,2) with adjustable power distribution. Line 1 path length was altered by translating mirrors M (1.1) and M (1.2) to synchronise the ablation spots temporally. This synchronisation is proven by the ablation interference patterns with Line 2 at the sample surface. The ablation spot separation was adjusted by slightly tilting mirror M (1.4). Both lines were focussed onto the sample surface by an *f* = 100 mm optic L2. At the focal plane, the laser spot radii are *w*_86_ = 15 µm. A spot monitor (CCD) on the level of the material surface to enable a precise temporal and geometrical adjustment. A probe laser (Cavilux, Fa. Cavitar Ltd., 688 nm/13 ns, Tampere, Finland) electronically synchronised to the pump laser allowed pump-probe shadowgraphy of expanding plasma plumes. The shadowgraphs were recorded with a 14-bit cooled CCD camera (pco.1600, PCO AG, Kelheim, Germany).

## 3. Results

### 3.1. 2-Spot Ablation with 10 ps Pulses at 1064 nm/5 kHz Repetition Rate

**Figure 3:**
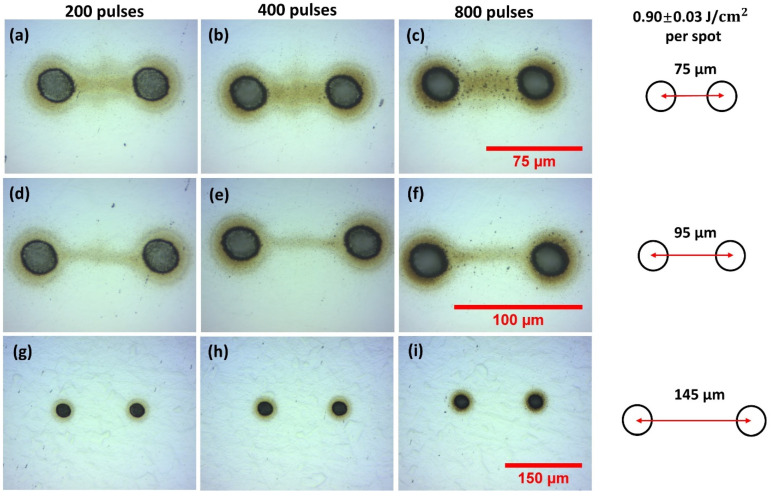
Optical images of observed two spot re-deposition during 10 ps laser ablation of stainless steel in air while varying spot separation and pulse number at F = 0.90 J cm^−2^, (**a**) 200 pulses, (**b**) 400 pulses, (**c**) 800 pulses, all 75 µm separation, (**d**–**f**) 95 µm separation, (**g**–**i**) 145 µm separation respectively. Debris is concentrated in a filament between the spots at 75 µm, thinning to a line about 10 µm wide at 95 µm spot separation. No interaction between the spots is observed at 145 µm separation at this low fluence.

The substrate used was ANSI 304 stainless steel and optically polished to a roughness Ra ~50 nm. The focussed single beam diameter was measured from the observed ablation crater diameters with increasing pulse energy [57] and found to be φ = 22.2 ± 0.2 µm. No significant variation with multi-spot geometry was found. Single-pulse ablation threshold (*N* = 1) was measured to be F_th_ = 0.29 ± 0.01 J cm^−2^ decreasing with pulse number, and incubation coefficient measured to be S = 0.85 ± 0.01 in excellent agreement with the literature [58]. Figure 3 shows the re-deposition patterns observed during two spot ablation with 10 ps laser ablation of stainless steel in air while varying separation and pulse number at fluence F = 0.90 J cm^−2^, (a) 200 pulses, (b) 400 pulses (c), 800 pulses, all 75 µm separation, (d)–(f) 95 µm separation, (g)–(i) and 145 µm separation, respectively. It required multi-hundred pulse exposure to observe debris for good optical contrast. The ablation debris is concentrated between the spots at *d* = 75 µm separation with a width comparable to the ablation spot diameter, while at 95 µm separation this narrows to around a 10 µm wide “filament”. The concentration of this directed re-deposition, observed at low fluence, is an interesting phenomenon, indicating transient forces during the plume expansion and collisions with the air, resulting in some ablation debris acquiring momentum components preferentially directed along the axis between the spots. There is evidence also that at the midplane, some debris is expanding normal to the axis. As spot separation increased to 145 µm at this fluence, there was negligible interaction between the plumes. The debris radius near spots R_d_ ~ 30 µm.

**Figure 4:**
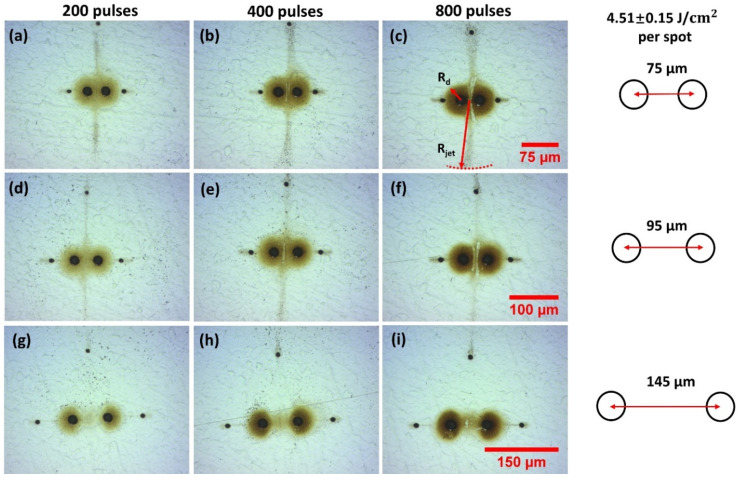
Optical images of observed two spot re-deposition during 10 ps laser ablation of stainless steel in air while varying spot separation and pulse number at fluence F = 4.51 J cm^−2^, (**a**) 200 pulses, (**b**) 400 pulses (**c**) 800 pulses, all 75 µm spot separation, (**d**–**f**) 95 µm separation, (**g**–**i**) 145 µm separation. At this higher fluence in (**a**–**c**), we observe diverging debris jets ejected normal to the spot axis while in (**c**,**f**), removal of surface debris by shock waves is apparent. These effects essentially disappear at the highest separation with a return to the concentration of material between the spots (**g**–**i**). The tiny ablation spots along the axis are due to low energy ghost beams while the top spot remains zero order.

The effect on debris re-deposition of increasing fluence to F = 4.51 J cm^−2^ on stainless steel is shown in the optical images of Figure 4a–i. At this higher fluence in Figure 4a–c, we observe diverging debris jets ejected normal to the spot axis. This extends to a radial distance of R_jet_ ~200 µm, well beyond that around each spot R_d_ ~ 30 µm, inferring that an energetic process may be involved. In Figure 4b,c, with increased exposure, removal of debris from the surface between the spots (in the form of two slightly curved lines) supports the view that strong shock wave interactions between the colliding plumes during ablation may be responsible, clear in Figure 4c,f. These effects essentially disappear at the highest, 145 µm separation with a return to the concentration of material between the spots Figure 4g–i, similar to the patterns at low fluence (Figure 3). The tiny ablation spots along the axis are due to low energy ghost beams appearing during multi-pulse exposure, while the top spot is the remaining zero order. Their presence here helps detect local physical effects of shock wave and air movements affecting the debris motion.

### 3.2. Ablation Rates and Debris Radii (10 ps Pulses)

**Figure 5:**
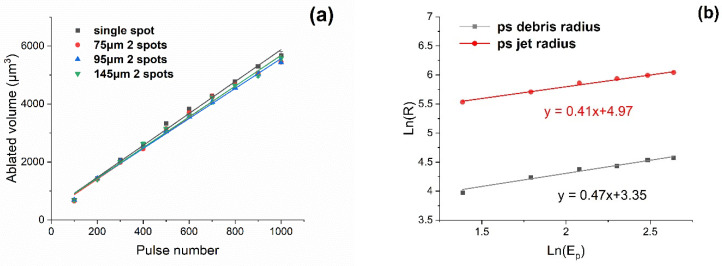
(**a**) Measured ablation volume versus pulse number for single and double spot ablation at *d* = 75 µm, 95 µm and 145 µm separation with fluence F = 2.9 J cm^−2^/spot (Ep = 6 µJ/spot). Spot geometry does not affect ablation volume for *N* ≥ 200, within experimental error, (**b**) Observed single spot debris radius and jet radius with pulse energy and exposure on an Ln-Ln plot. The radii follow a power-law R ∝ E^0.47^ for debris and R_jet_ ∝ E^0.41^ respectively.

The measured ablation volume/pulse of single and two spot geometries with pulse number N is shown in Figure 5a for a fluence F = 2.9 J cm^−2^ (E_p_ = 6 µJ/pulse). These results confirm that ablation rates are essentially independent of spot geometry and spot separation when *N* ≥ 200, whether single or double spot, within experimental error. Hence, the proximity of the spots does not affect ablation rates significantly. The ablation volume/pulse V ~ 5.7 µm^3^/pulse corresponding to a mass ablation M ~ 0.05 ng/pulse. Crater volumes and geometries were measured with a white light interferometer, Wyko NT3300. The single spot debris radius and jet radius with pulse energy and exposure are shown in Figure 5b on an Ln-Ln plot. This confirms that the debris radii follow a power-law R ∝ E^0.47^ for a single spot while the much higher Jet debris radii, R_jet_ ∝ E^0.41^. This relationship approximates that predicted by blast wave theory [59], however, higher than the expected R ∝ E^1/3^ which has also been observed by other authors [60].

### 3.3. SEM Imaging of the Debris Fields, Two Spot Patterns

**Figure 6:**
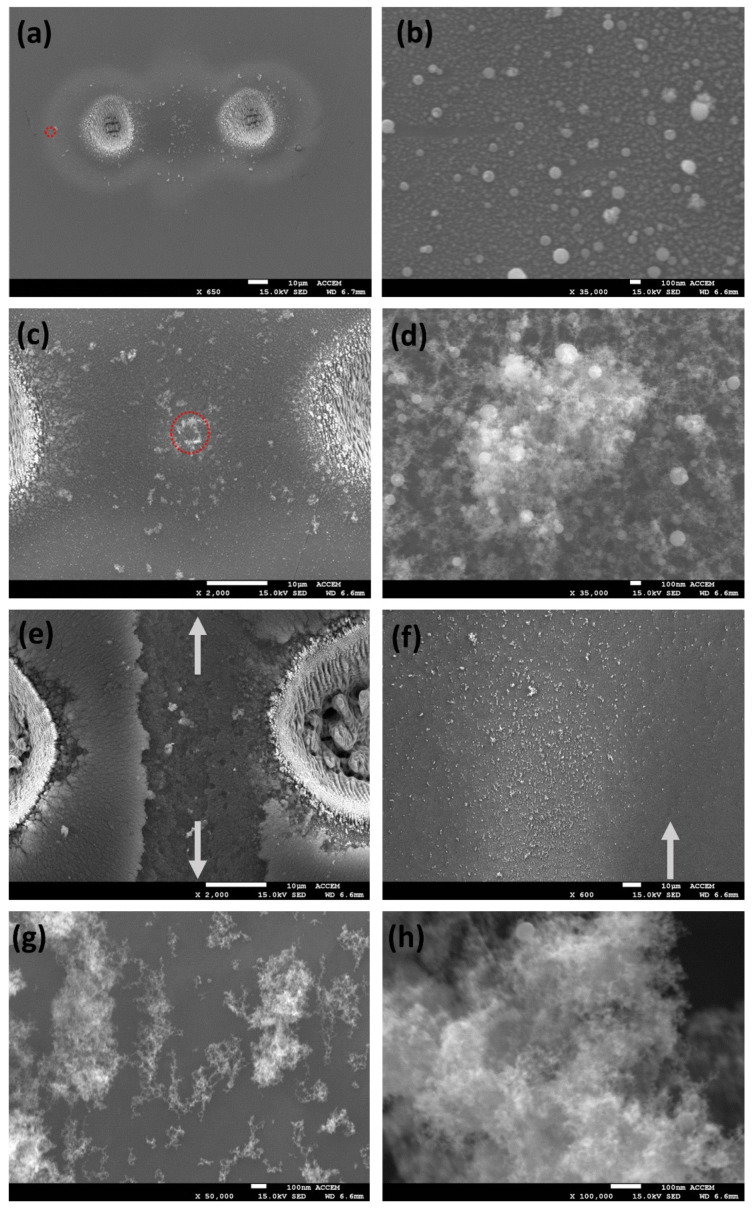
SEM images of the ablation debris from two spot ablation of stainless steel at low fluence (F = 0.9 J cm^−2^ (**a**–**d**) and high fluence F = 4.8 J cm^−^^2^ (**e**–**h**), *N* = 800 pulses, spot separation *d* = 75 µm. (**a**) image of the whole debris field, (**b**) 50–150 nm diameter np’s observed at spot periphery in (**a**) red ring, (**c**) particle agglomerate at the centre, (**d**) high magnification image of (**c**) showing round np’s and np agglomerate, (**e**) High fluence re-deposition pattern showing material lifted directly from the surface by the energetic colliding plumes and shock waves, (**f**) jet debris ejected normal to spots, (**g**) high magnification image of jet np chain agglomerate, (**h**) highest, 100,000× magnification of jet np chain agglomerate which still shows little structure.

Figure 6a–d shows a series of SEM images of ablation debris from two spot ablation of stainless steel in the air with 10 ps pulses at low fluence F = 0.9 J cm^−2^, *N* = 800 pulses and spot separation *d* = 75 µm. The concentration of debris between spots is clear in Figure 6a, with evidence of some material ejected normal to the spot axis in the centre. At low fluence, there are two main components to the debris—particle agglomerates appear on the collision plane (and around the spots), while solid spherical nanoparticles with diameters 50–150 nm appear at the spot edge, Figure 6b. These are likely formed during collisions of the expanding plumes and condensed from the stagnation region at the midplane. Figure 6c (2000×) shows the particle agglomerates near the centre at higher magnification while Figure 6d (35,000×) confirms that the particle agglomerate consists of both solid np’s and agglomerated chains of fine np’s. There is a significant change in the nature of the debris generated at higher fluence, F = 4.8 J cm^−2^, as shown in the SEM images of Figure 6e–h. Deposition now produces a thick deposit near the ablation spots while the shock waves lift material from the surface between the spots Figure 6e and deposit well away from the spots, Figure 6f. Figure 6g,h, with increasing magnification show that this jet debris consists of np chain agglomerates and is almost devoid of solid nanoparticles. This is consistent with higher surface temperature achieved during ablation, well above the evaporation temperature, T_ev_ = 2861 K. The high concentration of the np chain agglomerate debris supports the assertion that this material is formed during strong stagnation of the plume collisions in the midplane.

### 3.4. Time-Resolved Plasma Emission Spectra

**Figure 7:**
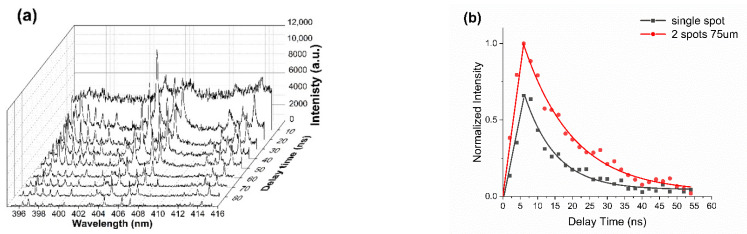
(**a**) Two spots (E = 20 µJ/spot, *d* = 75 µm,) time-resolved plasma emission spectrum (Δλ = 395–415 nm) of stainless steel over time delay 0–95 ns. Continuum dominates at early times while atomic lines appear as the plasma cools. The three intense lines near the centre are due to Fe I: 404.581 nm, Fe I: 406.359 nm, Fe I: 407.581 nm, (**b**) Integrated intensity (Δλ = 395 nm–415 nm) with time for single and double spot (*d* = 75 µm) showing exponential decrease with plasma lifetimes τ1/e ~ 9.2 ± 0.5 ns (single) and 13.9 ± 0.7 ns (double) respectively. The fits are exponential.

Figure 7 shows the two spots (E = 20 µJ/spot, *d* = 75 µm) time-resolved (0–95 ns) plasma emission spectrum of stainless steel in air over Δλ = 395 nm–415 nm. Gate width was set at 5 ns, gate delay interval 2 ns and data accumulated over 50 spectra. The substrate was scanned at 2 mm/s while an f = 125 mm bi-convex lens imaged the whole plasma emission to a fibre coupler (NA ~0.2, Figure 1) then to the spectrometer (Andor Shamrock 303i, 50 µm slit, 1800 L/mm grating) and cooled ICCD camera. The ICCD was triggered externally from the Laser Regenerative amplifier. Continuum dominated the spectra at early times, likely black body radiation from the hot plasma near the surface [21], while spectral line intensities rise sharply, then decrease along with the background continuum as the ablation plasma cools. Spectral line widths also decrease with time as electron density decreases, reducing Stark broadening [61]. The spectral lines in this region have been identified as due mainly to excited neutral atoms of Fe I, Cr I and Mn I: Fe I: 395.667 nm, Cr I: 396.368 nm, Cr I: 396.974 nm, Mn I: 397.708 nm, Fe I: 398.396 nm, Mn I: 399.161 nm, Fe I/Fe II blended line: 403.130 nm, Mn I: 403.307 nm, Fe I: 404.581 nm, Fe I: 406.359 nm, Fe I: 407.581 nm (centre), Fe I: 411.854 nm, Fe I: 413.290 nm, Fe I/Fe II/Fe III blended line: 414.26 nm. Single spot (20 µJ/pulse) spectra were very similar with lower intensities [62].

Figure 7b shows the time-integrated plasma emission (Δλ = 395–415 nm) for single and double spot (*d* = 75 µm) confirming that the plasma lifetimes τ_1/e_ ~ 9.2 ± 1.0 ns and 13.9 ± 0.7 ns, respectively, hence double spot plasma lifetime increasing over the single spot. These lifetimes are similar to those observed with 20 µJ, 150 fs laser ablation of Al in the air [21]. The short plasma lifetimes are a consequence of both the low pulse energies and rapid collisional cooling of the dense ambient air.

Plasma excitation temperature for single spot ablation (E = 20 µJ) has been estimated by the well-known Boltzmann method [11] from the Fe I line intensities I_mn_, transition probabilities (gA_mn_, g degeneracy) and upper energy levels, E_m_. A plot of Ln(λI_mn_/gA_mn_) versus E_m_ yielded a linear plot inferring T_e_ ~7500 K for single spot near 40 ns delay time. We also estimated the electron density from the Stark broadening of the Fe I line at λ = 404.58 nm yielding N_e_ ~ 10^18^ cm^−^^3^.

**Figure 8:**
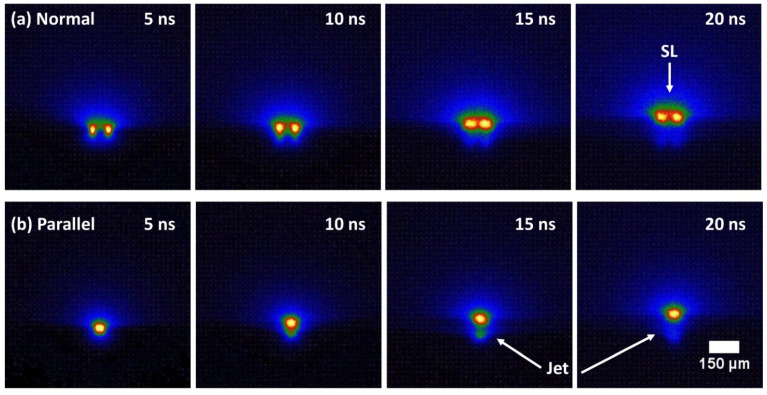
Time-resolved imaging of single and double spot ablation plume emission (20 µJ/spot, *d* = 75 µm) of stainless steel with time delays 5–20 ns. ICCD gate width was 5 ns (**a**) 2-spot normal to optic axis showing plume collisions and stagnation at 20 ns delay (**b**) two spots parallel to optic axis with an indication of jet structure 15–20 ns.

By removing the ICCD from the spectrometer and placing this at the image plane of the focus lens, (f = 75 mm, M ~ 4), the time-resolved plasma plumes were observed, Figure 8. Gate width here is 5 ns, energy/spot = 20 µJ and spot separation *d* = 75 µm. With spots normal to the optic axis, we can observe plasma expansion, collision and stagnation after 15 ns. When the plumes are imaged parallel to the optic axis and 10–15 ns delay, we see some interesting structure at right angles to the spot axis which may be connected to the jets. The lateral plume expansion velocity can be estimated to be v_⊥_ ~3.5 × 10^3^ ms^−1^, decelerating after 15 ns, while the elliptical plume shape which develops supports lateral plasma expansion [35,63].

### 3.5. Two Spot Ablation with 600 fs/5 kHz Temporal Pulses at 1030 nm

**Figure 9:**
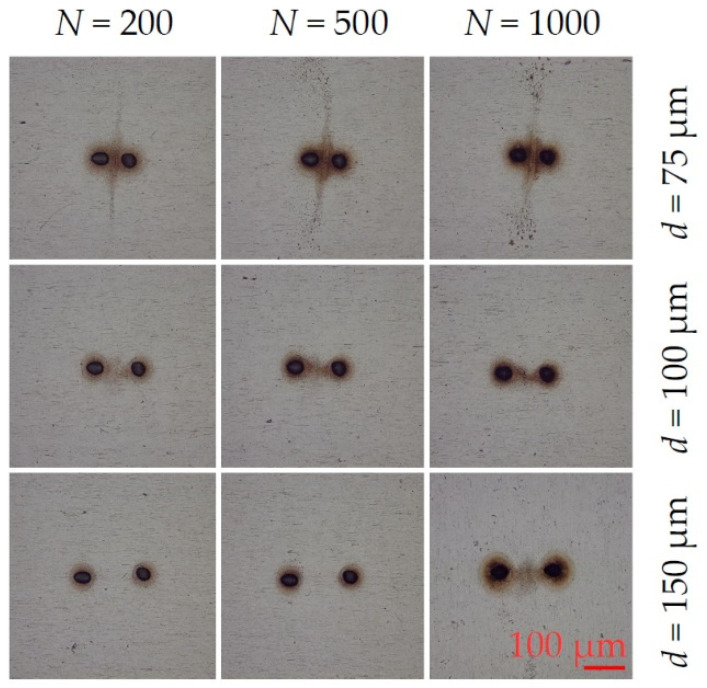
Resulting debris re-deposition and jet formation after 600 fs laser double pulse ablation with fluence F_0_ = 1.41 J cm^−2^ (E_P_ = 5 µJ/pulse). Optical images of stainless steel surfaces after applying for various pulse numbers (200, 500, 1000) at different spot separations (50 µm, 100 µm and 150 µm). Debris jets are visible at 75 µm at the investigated *N*. At larger separations, the debris is concentrated in a filament between the spots (*d* = 100 µm) and becomes apparent at *d* = 150 µm separation, *N* = 1000. Fluence here is 1.5 times higher than with 10 ps pulses (Figure 3).

The effect of increasing peak intensity by over 1 order of magnitude was investigated with the experimental system of Figure 2, while maintaining the same repetition rate of 5 kHz. Results are shown in Figure 9 for pulse numbers 200, 500 and 1000 at fluence F = 1.41 J cm^−2^ (peak intensity I = 2.4 × 10^12^ W cm^−2^). Again, we observe symmetric debris ejection or jets at right angles out to a radius of R_jet_ ~ 150 µm with spot separation *d* = 75 µm. As spot separation increases, we return to the directed, filamentary re-deposition (*d* = 100 µm) between the spots, and just apparent at *d* = 150 µm, *N* = 1000), similar to patterns observed with 10 ps pulses, Figure 3a–f.

**Figure 10:**
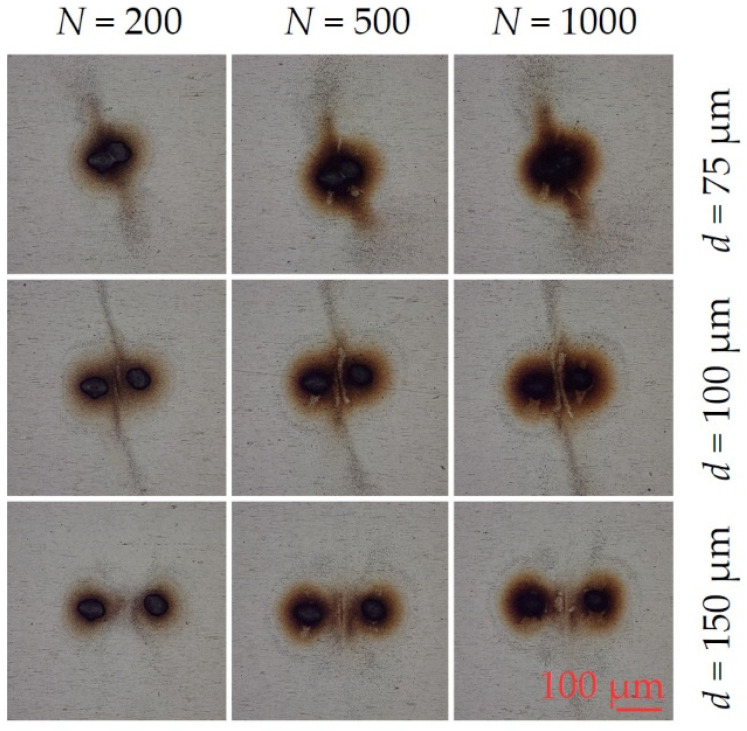
Resulting debris re-deposition and jet formation after 600 fs laser double spot ablation with fluence F_0_ = 5.7 J cm^−2^ (E_P_ = 20 µJ/pulse). Within the investigated pulse numbers *N* (200, 500, 1000) and pulse separations *d* of 50 µm, 100 µm and 150 µm, the optical images reveal an interaction between the two spots. Strongly diverging debris jets at 75 µm and collimated jets at *d* = 100 µm are visible. At the largest separation *d* = 150 µm, where the interactions are weaker, the material is again concentrated between spots.

The results of high fluence two spot ablation with fluence F = 5.7 J cm^−2^ (20 µJ/spot, I = 9.6 × 10^12^ W cm^−2^) are shown in Figure 10. where plume interactions create strongly diverging jets at spot separation *d* = 75 µm. At *d* = 100 µm separation, jets are more collimated with re-deposition jet radius R_jet_ > 300 µm. There is a slight tilt in the spot axis here relative to the horizontal. With *N* = 500 and 1000 at *d* = 100 µm, shock wave and plume interactions also remove debris from the surface (compare Figure 4c,f), evidence of the quasi-stationary shock waves [51,52]. At *d* = 150 µm material is again concentrated between the spots as interactions weaken. Peak intensity therefore appears to play a minor role in the debris re-deposition during the plume interactions from 600 fs to 10 ps pulse length on stainless steel in the air.

**Figure 11:**
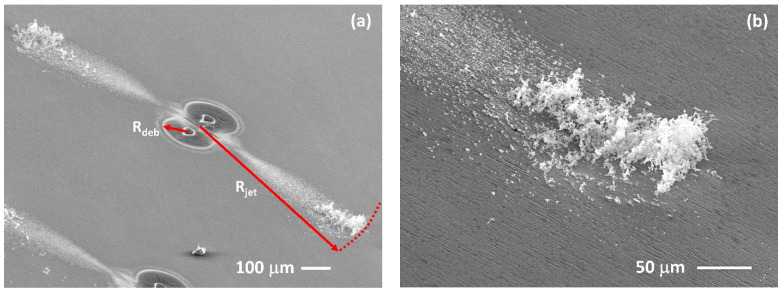
Tilted SEM images of the jets after double spot femtosecond ablation of s. steel in the air (E_P_ = 20 µJ/pulse, *N* = 1000, *d* = 100 µm). (**a**) shows the symmetric diverging debris jets (np chain agglomerates) with an accretion of material at the jet ends, almost 400 µm from the double spot centre. (**b**) higher magnification of concentrated jet debris near the jet end. The debris radii near the spots and jet radii are indicated (R_deb_ and R_jet_ respectively).

Figure 11a,b show SEM images of the diverging symmetric jet debris from 600 fs double spot (*d* = 100 µm) ablation of stainless steel in air (20 µJ/spot, F = 4.5 J cm^−2^, *N* = 1000). This material likely consists of np chain and is concentrated at the jet ends, almost 400 µm from the spots.

**Figure 12:**
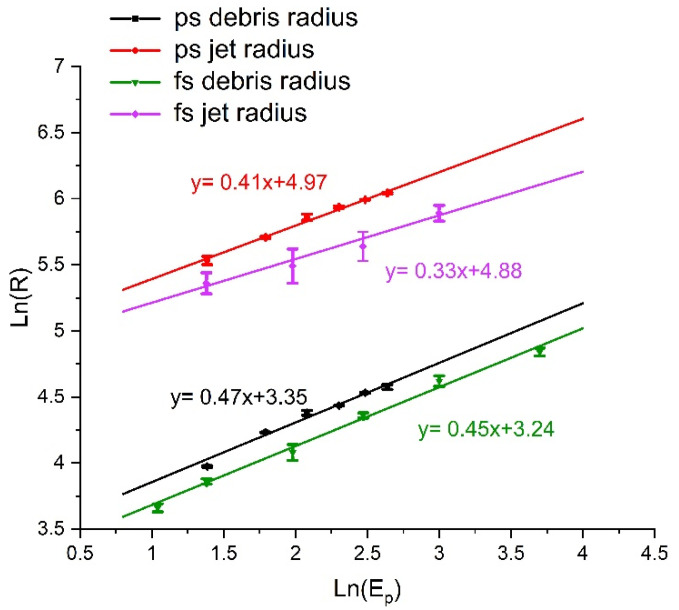
Ln-Ln plots of the two spots debris and jet radii ([R] = µm) measured for 600 fs and 10 ps ablation. The logarithmic fits ([E_P_] = µJ) are shown to be reasonably close in exponent except for femtosecond ablation which is lower. The spot separations were 100 µm (600 fs) and 75 µm (10 ps), respectively. Error bars represent 1 σ.

The debris and jet radii generated with 600 fs pulses are shown in Figure 12. For comparison, the data from 10 ps two spot ablation. Logarithmic fits are included, and the exponents vary from n = 0.33 to 0.47. The fs and ps debris radii are close exponent while the fs jet radii appear to have the lowest, n = 0.33. The higher radii for given energy with 10 ps pulses is due to the smaller spot separation of 75 µm, while this was 100 µm with 600 fs pulses. There may also be differences due to uncertainties in the estimation of the radii.

The reproducibility of the results reported is supported by the excellent observed fits to the Ln-Ln plots with indicated errors. Ultrafast laser ablation minimises thermal diffusion and melt leading to a deterministic evaporation process [19]. The observed nanoparticle agglomerated jets can be deflected with a significant airflow over the substrate. However, when the surrounding ambient air is stable, the jets emanate at right angles and have the same length, Figure 11a.

### 3.6. Time-Resolved Shadowgraphy (600 fs/5 kHz).

**Figure 13:**
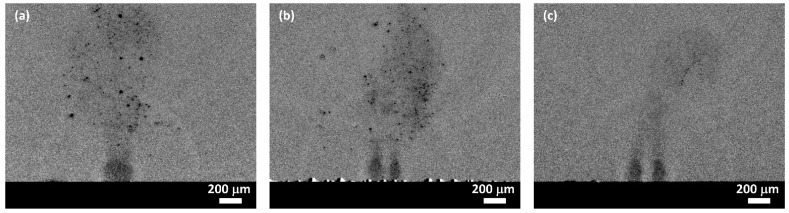
Time-resolved shadowgraphs of the interacting plumes with two spot ablation in the air after *N* = 6 and delay time of τ = 2 µs after ablation and τ (total) = 1002 µs. These images demonstrate that large condensed absorbing particles are apparent in the debris above the spots when the separation is smallest and where the plume collisions are strongest, (**a**) *d* = 100 µm, (**b**) *d* = 150 µm, (**c**) *d* = 200 µm where these particles have all but disappeared. Note that the spherical shock waves from the last pulse overlap the material ejected beforehand.

Ablation plumes and their development after ablation were imaged with the pump-probe experimental set-up shown in Figure 2. The probe beam (*λ* = 688 nm, *τ*_H_ = 13 ns) was electronically synchronised to the pump beam (*λ* = 1030 nm). Time-resolved measurements are often carried out with a single pulse exposure–but here, after simultaneous multi-pulse, multi-spot exposure, we can see the developing plumes and their interactions. The connection axis of two spots is aligned perpendicular to the imaging plane. The total delay time regarding the arrival of the first pulse pair with 5 kHz repetition rate is τ = 1002 µs, hence a delay time of 2 µs after the last, *N* = 6th pulse, Figure 13. There is a strong plasma plume overlap confined to the surface at *d* = 100 µm separation with diverging plume above the spots containing solid (dark) micron size particles, strongly absorbing/scattering. The previous pulses have formed these. These also appear at 150 µm spot separation but disappear entirely at *d* = 200 µm, where the plasma plumes near the surface are distinct and plume absorption much more uniform. This particle is likely the np chain agglomerate observed on the surface, shown in the SEM images of Figure 11. which appear in the jets due to the strong plume interactions. Note in the shadowgraphs a visible expanding spherical shock wave from the last pulse pair overlaps the material previously ejected. The shock wave speed, from the time delay, *v*_s_ ~375 m/s just above the speed of sound while the lateral plasma expansion has slowed significantly to around *v* ≤ 50 m/s after 2 µs delay.

## 4. Discussion

Laser ablation on metal in the ambient atmosphere can be likened to a mechanical detonation, creating an ablation plume expanding supersonically against the background gas, causing a shock wave. As the plume does work by expanding against atmospheric pressure, the expansion velocity decreases with time, and the shock wave radius is given by Taylor’s blast wave theory [59],

(1) $$R=S\left(\gamma \right){\left(E/\rho_{0}\right)}^{1/5}t^{2/5}$$

where *S*(*γ*) ~1 is a function only of the air specific heat *γ* (~1.4), *E* is the energy released, *ρ*_0_ is the undisturbed background gas density and t is the time after ablation. The effect is to force most of the air within the shock front into a thin shell just inside the front, compressing and heating the air. The initial pressure driving the front *p*_max_ ≫ *p*_0_, (1 atm). When *p*_max_ ~ *p*_0_, the self-similar solution of Equation (1) is no longer valid. In this case, the radius for this upper limit is given by [20],

(2) $$R={\left(E/p_{0}\right)}^{1/3}$$

The ablation plasma plume follows behind the shock front and a contact discontinuity can be observed inside the shock front [33]. For the range 2 µJ ≤ *E* ≤ 20 µJ, single spot debris radii range from 40–120 µm while *R* calculated from Equation (2) yields 270 µm ≤ *R* ≤ 580 µm. The debris radius can be related to the shock radius through R_D_ ~ fR where f < 1 and here, f ~ 0.15–0.2, similar to that observed by other authors [60]. From the time-resolved plasma emission (10 ps), backward re-deposition with single spot ablation likely starts about ~100 ns after ablation and from the pump-probe observations, (600 fs) continues over 10 µs and longer. The debris power laws yield R_deb_ ∝ E^0.3–0.5^ for both single spot and jets but the much larger jet radii if applied to R_D_ ~ fR yields f ~ 0.7–0.8 using Equation (2) inferring that an additional energetic process is involved during the plasma plume interactions.

Multi-spot ablation in vacuum between two independent and closely spaced seed plasmas collide due to the lateral plasma expansion. With a low density and high relative velocity, plasmas tend to interpenetrate, relevant in collisionless astrophysical plasmas, leading to ion reflection and particle acceleration [64]. However, with higher density and a low relative velocity, the plasmas rapidly decelerate at the collision plane, forming a stagnation layer (SL). Accretion and compression of the material within the SL leads to a local increase in density and temperature. The degree of stagnation can be described by a collisionality parameter *ξ* = *d*/*λ_ii_*, where *d* is the distance between the two plasmas and *λ_ii_* is the ion-other ion mean free path, given by, [65,66]

(3) $$\lambda_{ii\;\left(1-2\right)}=\frac{4\pi \varepsilon_{0}^{2}m_{i}^{2}v_{12}^{4}}{\;q^{4}Z^{4}n_{i}\ln\left(\Lambda_{1-2}\right)}$$

where *ε*_0_ is the permittivity of free space, m_i_ is the ion mass, $v_{12}$ is the relative ion flow velocity (prior to impact), *q* is the elementary charge, *Z* is the average ionisation state, *n_i_* is the plasma density at the collision plane and ln (Λ_1–2_) is the Coulomb Logarithm, a slowly varying function, with a value *O* (10–20) [67]. The parameter *ξ* is very sensitive to the relative plasma velocity term $v_{12}$^4^ while only linearly dependent on separation d. If used in ambient air, this description is more complex than in vacuum due to the presence of shock waves and rapid plasma deceleration due to collisions with the air. From time-resolved plasma emission at 15 ns, Figure 8. We estimated that *v*_⊥_ ~ 3.5 × 10^3^ m s^−1^ so that $v_{12}$ = 2*v*_⊥_ ~ 7 × 10^3^ m s^−1^. Inserting this value into Equation (3), we find *λ_ii_* ~ 3.1 µm so that the plasma collision region is limited to the micron scale with collisionality parameter *ξ* = *d*/*λ_ii_*= 75 µm/3.1 µm ~ 24.

In a plasma, the Debye length is the characteristic length over which electrons and ions can be separated, and in an ideal plasma (e.g., astrophysical) has many particles per Debye sphere *N_D_* given by

(4) $$N_{D}=N_{e}\left(\frac{4\pi }{3}\right)\lambda_{D}^{3}\gg 1$$

The classical plasma parameter g = 1/*N_e_λ_D_*^3^ << 1 so that collective effects dominate the plasma. In laser ablation, we can calculate g for each plasma plume knowing *N_e_* and *λ_D_* which is given by [67] *λ_D_* = 743 (T_e_)^1/2^ (*N_e_*)^−1/2^ where the electron temperature T_e_ is in eV while *N_e_* is in cm^−3^. At delay time τ = 50 ns, we measured T_e_ (10 ps, E_p_ = 20 µJ) from a Boltzmann plot to be ~7500 K (1 eV = 11,600 K) hence plasma temperature T_e_ ~ 0.65 eV while *N_e_* ~ *N_i_* ~ 10^18^ cm^−3^ from measured Stark broadening of the Fe I line at *λ* = 404.58 nm. The Debye length in each plasma plume is then *λ_D_* ~ 6.0 × 10^−7^ cm (6 nm) while the particle number in a Debye sphere is, from Equation (4), *N_D_* = 0.92. The collision parameter g = 1/*N_e_λ_D_*^3^ = 4.6 hence the plasma plumes are each highly collisional as expected. We can also estimate the Coulomb Logarithm [67], LnΛ = 9*N_D_*/*Z* = 8.3 assuming *Z* ~ 1 is the average ionisation state, supported by the time-resolved spectroscopy, Figure 7a.

Recently, the interaction of two colliding Al plasmas (in the air) and their shock waves were observed in two spot nanosecond laser ablation using shadowgraphy, schlieren images and interferometry [68]. From refractive index profiles, expanding shock fronts and their reflections were observed after collision along with compression and stagnation and of the air behind the shock fronts. With pulse fluence F ~ 27 J cm^−2^, focus intensity I ~ 1.6 GWcm^−2^ and 1 mm spot separation, compressed air density reached *n*_0_ ~ 5 × 10^20^ cm^−3^ behind the interacting shocks while plasma density *n_i_* ~ 5.10^18^ cm^−3^. The stagnation was described as “soft” [69] with a plasma hill developing over times scales from 0.7–3 µs.

More recently, stationary transient straight shockwaves (on a 10 ns timescale) were detected during multi-spot fs ablation of water in the air creating supersonic air flows which collided [51,52]. Fluence per spot F ~ 18 J cm^−2^ with peak intensities I ~ 10^14^ W cm^−2^ and spot separations from d ~ 14–20 µm. The length of the shock waves was related to the local flow velocity at a given radial position from the spots and observed when the relative speed of shock wave propagation exceeded the velocity of sound in the air. The symmetric geometry of the observed two spot transient shock waves is highly reminiscent of the observed patterns of debris removed between double spot ablation in this work at higher fluence (Figure 4, Figure 6 and Figure 11) with ps and fs pulse lengths. This indicates that material removal between spots during ablation at high fluence may be evidence of shock wave interactions.

## 5. Conclusions

**Figure 14:**
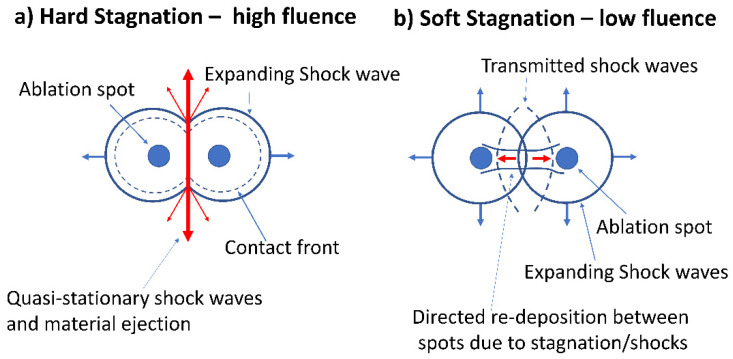
Schematic diagrams summarising current view of two spot ablation at high and low ablation (**a**) at high fluence, the air between the shocks is compressed by a “piston” as they meet and the curved shocks will accelerate and expel the air symmetrically in a diverging jet in both directions normal to the axis. The plasmas then stagnate and Coulomb interactions off-axis at low impact parameters convert their axial momentum to transverse momentum, (**b**) at low fluence and larger separations, shock wave pressures are much reduced, plasma density decreases, reducing stagnation to “soft”, hence allowing plasma interpenetration and directed deposition between the spots.

The jets appearing at higher fluence (and small spot separation) could be created as follows using a simplified 2D approach. When the high-pressure shock waves meet at the collision plane, the pressure due to each shock wave *p*_max_ = 0.155 E/R^3^ (*γ* = 1.4) [59]. If we set E ~ E_p_ = 20 µJ and R = 50 µm, *p*_max_ = 2.48 × 10^7^ Pa ~248 atm. The air between these shocks will be highly compressed by this “piston” as they meet and the curved expanding shocks could compress, accelerate and expel the air symmetrically in a diverging jet in both directions normal to the axis, Figure 14a. The plasmas following then stagnate and ion Coulomb repulsion off-axis at low impact parameters converts their axial momentum to transverse momentum assuming elastic collisions. The observed material lifted from the surface leaves patterns very similar to the transient stationary shock waves observed during multi-spot induced supersonic air flows at a water/air interface [51,52].

At low fluence and larger separations, shock wave pressures are much reduced, plasma density decreases, reducing stagnation to “soft”, hence allowing plasma interpenetration between the spots. Weak shock waves pass through each other and likely reflect back along the axis from the plasmas. Multiple collisions can decelerate ions and a degree of ion reflection may occur [64] so that particles acquire momentum directed along the spot axis then arrive at the surface through backward flux, Figure 14b.

Two colliding air breakdown plasmas created with high energy, nanosecond laser pulses demonstrated reflection and transmission of expanding shock waves depending on pulse energy and plasma seed separations [70]. One might ask if oxidation during ultrafast laser ablation is significant here, but we have also observed the Jets in pure Nitrogen at 1 atm (10 ps, not reported here) with the aid of a gas cell. We estimate that oxidation of Fe atoms to Fe_3_O_4_ could at most add 10% additional energy, not nearly enough to explain the phenomena here.

Time-resolved plasma emission yielded important plasma parameters such as lifetime and temperatures, and plasma collisions between spots show plume stagnation, resulting in bi-directional jets. The effects of quasi-stationary shock waves are very clear, compressing the air and lifting debris from the surface between the spots. Time-resolved shadowgraphy with 600 fs pulses showed micron size particles (which constitute the jets) only when the ablation spots were in close proximity. In fact, the re-deposited jet debris consists of agglomerated nano-particle chains.

Our observations of the directed deposition at low fluence and jets during high-fluence, multi-spot ultrafast laser ablation of stainless steel in air constitute a potentially interesting new area of research, providing a basis for further investigations to build a thorough understanding of these complex phenomena. Our study and its findings are considered important for a deeper understanding of the interaction of multi-spot arrays in laser patterning [71,72] and enhanced laser deposition of thin films [53,55]. The phenomena of plasma collisions and shocks observed and explored here should also be relevant to astrophysics and physics research, such as in magnetic re-connection [73] and phenomena such as bi-directional jet formation and particle acceleration [74,75] observed at ultrahigh laser intensities (10^14^–10^15^ W cm^−2^) in two spot ablation with high transient B fields.

## Data Availability

The data presented in this study are available on request from the corresponding author.
